# Supplementary material for: Analytical Model of CVD Growth of Graphene on Cu(111) Surface
Source: Nanomaterials (Basel). 2022 Aug 27;12(17):2963. doi: 10.3390/nano12172963 (PMC9457873; doi:10.3390/nano12172963)
Supplement: Supplementary file 1 [file nanomaterials-12-02963-s001.zip › nanomaterials-1878722-supplementary.pdf]

Supplementary Materials: Analytical model of CVD growth of graphene on Cu (111) surface

Ilya Popov [1,†,‡], Patrick Bügel [2,‡], Mariana Kozlowska [2], Karin Fink [2], Felix Studt [1,3] and Dmitry I. Sharapa [1*]

1. DFT calculations and Reaction rates

The core electrons were described by the projector augmented wave (PAW) method [1]. For the plane wave expansion, a kinetic energy cutoff of 450 eV and an energy convergence threshold of 10^{-6} were used. The optimized bulk lattice constants of 3.56 Å for the PBE-D3 functional and 3.64 Å for the BEEF-vdW functional were used throughout all calculations. The upper layers and adsorbed hydrocarbon species were relaxed with the conjugate gradient method until the change in the total energy between two ionic relaxations was smaller than 10^{-5}. The Brillouin zone was sampled by a 6 × 6 × 1 and a 2 × 6 × 1 Monkhorst–Pack k-point mesh for the 3 × 3 slab and 10 × 3 slab, respectively. Transition states were located with the nudge elastic band (NEB) method [2]. All calculations were conducted according to the non-spin-polarized scheme.

With respect to the reactions of small species, Li et al. [3] used a four-layer 4 × 4 slab with a 4 × 4 × 1 Monkhorst–Pack k-point mesh where only the bottom layer was frozen. Moreover, they employed an energy cutoff of 400 eV throughout their calculations. The different setups had only a small influence on the found barriers with the exception of reactions where subsurface carbon was involved, since we did not include subsurface carbon in our calculations.

More significant changes had taken place in the part of ribbon attachments, and the attachment of C_2 (as a particle proclaimed to be the dominant feeding species) is the most prominent case. Here, Li et al. was using a 7 × 4 slab with a 2 × 4 × 1 Monkhorst–Pack k-point mesh. They found a barrier of 0.58 eV (2.19 eV for the detachment barrier) on the model where two-ring-thick graphene ribbons were separated by less than 9 nm (Figure S1).

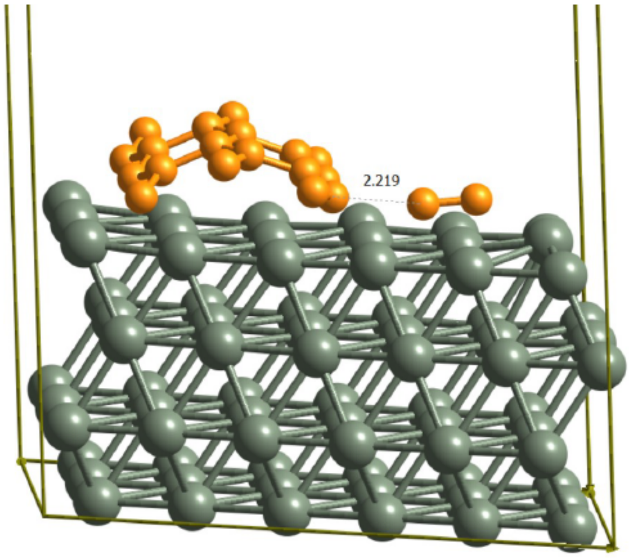

Figure S1. Transition state for the attachment of C_2 to the graphene zigzag edges on the Cu(111) that was found by Li et al. [3].

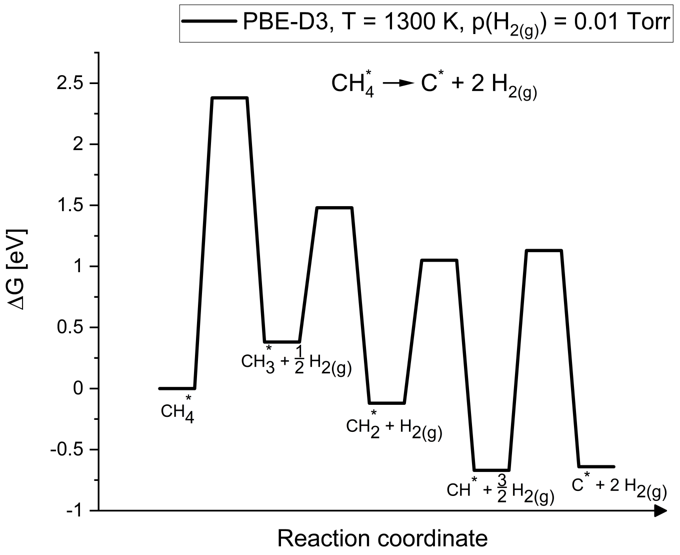

Figure S2. Gibbs free energy diagram of $CH_4^* \rightarrow C^* + 2H_{2(g)}$ process.

Table S1. Comparison of diffusion and formation barriers (PBE-D3) in eV for carbon monomer, dimer, and trimer. Values in brackets are taken from [4], where they were calculated with PBE.

Specie	Diffusion (eV)	Formation (eV)
C	0.15 [0.06] [a]	-
C_2	0.48 [0.49]	0.55 [b] [0.3]
C_3	0.19 [0.35]	0.85 [1.15]

[a] Diffusion between two on-surface minima. In [4], a lot of attention is dedicated to subsurface carbons.
[b] With BEEF-vdW, we obtained barrier of 0.25 eV; see Table 2 of main text.

Despite the diffusion barrier of dimer (0.48 eV) being higher than kT (which under this temperature would be around 0.11 eV), the lattice gas model is applicable for the case, as attachment-to-edge barriers are much higher and the equilibration of the surface densities in all points was reached faster than the reaction. Mathematically, it is shown in particular in Equation 10 of [5]. When D_s is significantly bigger than β, one can neglect the second term of the denominator and significantly simplify the equation. In our case, 0.73 eV (the difference between dimer diffusion and attachment) going to the exponent results in 4-5 orders of magnitude domination of the first term and thus J becomes independent from the diffusion.

Table S2. Equilibrium concentration c_{eq} of C and C_2.

Specie	Equilibrium concentration c_{eq} in monolayers
C	$2.67 \cdot 10^{-5}$
C_2	$4.87 \cdot 10^{-5}$

2. Details of solving kinetic equations from the main text

To transform the Fokker–Planck equation with the growth rate in Eq. (7) of the main text to the canonical form, we introduce a new variable $\rho = \sqrt{n}$. Its substitution gives the following:

$$\frac{\partial g(\rho,t)}{\partial t} + \frac{1}{2} v(\rho,t) \frac{\partial g(\rho,t)}{\partial \rho} = 0 \tag{S1}$$

Additional equations required to complete the system are obtained by expressing the rates of C and C_2 production/consumption as

$$\frac{d\xi_C}{dt} = \frac{J}{c_{eq}(C)} - 2k_d c_{eq}(C)(1+\xi_C)^2 - \frac{2\xi_C}{c_{eq}(C)t_1}\int_0^\infty \rho g(\rho,t)d\rho \tag{S2}$$

$$\frac{d\xi_{C_2}}{dt} = \frac{k_d c_{eq}^2(C)}{c_{eq}(C_2)}(1+\xi_C)^2 - \frac{2\xi_{C_2}}{c_{eq}(C_2)t_2}\int_0^\infty \rho g(\rho,t)d\rho \tag{S3}$$

Note that the lower limit of integration in the last terms of these equations is set to 0 instead of $\sqrt{n_*}$, which corresponds to an assumption that the sizes of growing nuclei which we are interested in (typically a few μm) are much higher than the chosen boundary n_*. This assumption is adequate for our system and allows us to exclude the value of n_* (the choice of which is associated with a known uncertainty) from the solution.

Equation (12) (main text—S3) has to be solved for the functions $\xi_C(t)$, $\xi_{C_2}(t)$, $\xi(t)$, and $g(\rho,t)$, which can be done by introducing the operators P_1,P_2, and P_3:

$$P_1(\varphi(t)) = \frac{d}{dt}\left(\frac{t_1\varphi(t)}{\xi_C(t)}\right) \tag{S4}$$

$$P_2(\varphi(t)) = \frac{d}{dt}\left(\frac{t_2\varphi(t)}{\xi_{C_2}(t)}\right) \tag{S5}$$

$$P_3(\varphi(t)) = \frac{d}{dt}\left(\frac{t_1\varphi(t)}{\xi(t)}\right) \tag{S6}$$

This allows us to transform integro-differential eqs. (S2-S3) into a system of two second-order entangled differential equations for the oversaturation profiles, which can then be solved numerically. Looking ahead, we can note, however, that for C concentration one can fairly apply the quasistationary assumption since in our conditions it is a highly reactive particle with a rather low production rate. This allows for a significant simplification of the equations for the oversaturation profiles, which yields the following for effective oversaturation:

$$P_3^2\left(1 - \frac{t_2 c_{eq}(C_2)}{t_1 J}\xi'(t)\right) = \frac{2I(\xi(t))}{J} \tag{S7}$$

3. Derivation of expressions for the rate constants

3.1. Carbon adatoms production rate

Let us consider the step-wise catalytic decomposition of methane on the metal surface following the scheme

$$CH_{4(g)} \rightleftharpoons CH_4^* \qquad (r_{ads}, r_{des})$$

$$CH_4^* \rightarrow CH_3^* + H^* \qquad \left(r_{dis}^{(1)}\right)$$

$$CH_3^* \rightarrow CH_2^* + H^* \qquad \left(r_{dis}^{(2)}\right)$$

$$CH_2^* \rightarrow CH^* + H^* \qquad \left(r_{dis}^{(3)}\right)$$

$$CH^* \rightarrow C^* + H^* \qquad \left(r_{dis}^{(4)}\right)$$

where r_{ads} and r_{des} are methane adsorption and desorption rates, $r_{dis}^{(i)}$ are the dissociation rates of the surface CH_x^* particles, and $*$ marks the particles adsorbed on the surface.

As it was discussed in the main text, the proposed model concentrates on the roles of C and C_2 in nucleation and growth processes, while CH_x^* (x $= 1 \ldots 3$) particles' impact is ne-

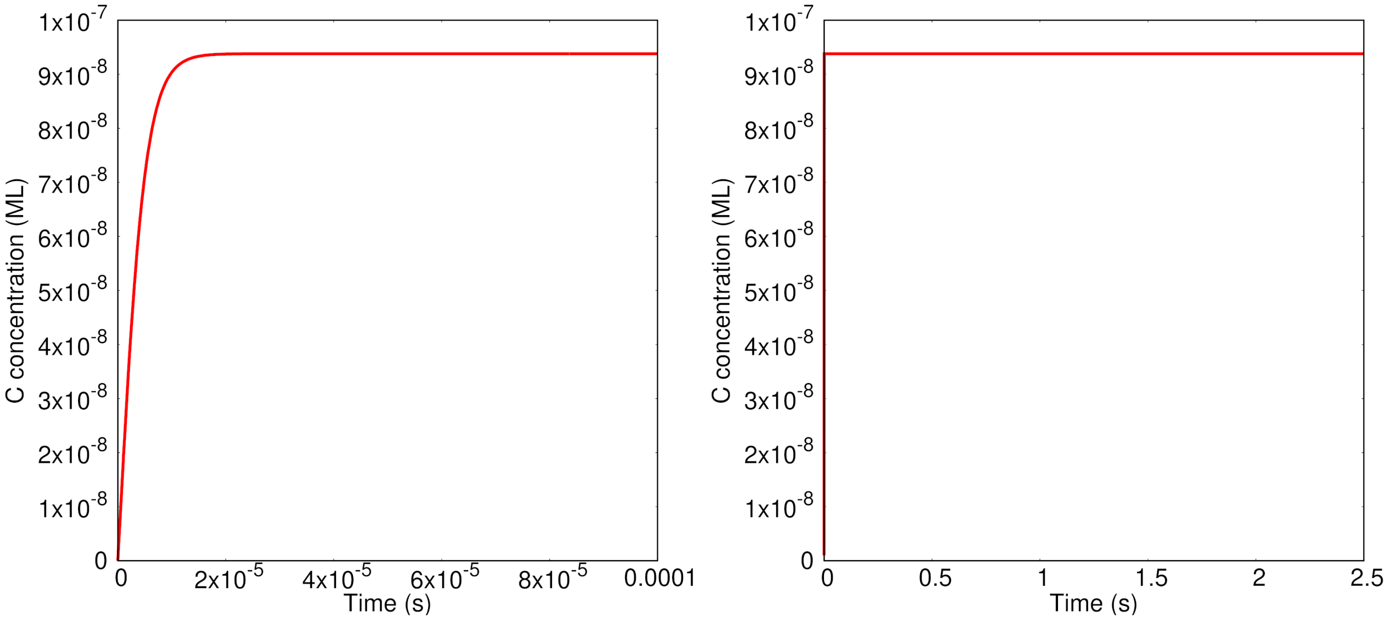

Figure S3. C concentrations as functions of time: left—on the initial stage; right—in the general timescale of the process.

glected and their concentrations are assumed to satisfy the quasistationary approximation. The latter assumption is supported by KMC calculations (Figure S4 of [3]), where it was shown that CH_x^* concentration profiles reach a stationary regime during graphene growth. From the quasistationary approximation ($dc(CH_x^*)/dt \approx 0$), one obtains the following relations between dissociation rates: $r_{dis}^{(1)} \approx r_{dis}^{(2)} \approx r_{dis}^{(3)} \approx r_{dis}^{(4)}$. Therefore, the rate of C adatoms production can be calculated as

$$J = \frac{dc(C^*)}{dt} \approx r_{dis}^{(1)} = \nu_0 \exp\left(-\frac{E_{dis}}{RT}\right) c(CH_4^*) \quad (S8)$$

where E_{dis} is the methane dissociation barrier and ν_0 is the standard frequency. The surface concentration of methane adparticles ($c(CH_4^*)$), entering this expression, has to be related to methane pressure in the gas phase usually known and controlled in experiment. This can be accomplished if we assume Henry's law for methane adsorption, which implies dynamic equilibrium between adsorption and desorption rates ($r_{ads} = r_{des}$).

The rate of methane adsorption can be calculated as

$$r_{ads} = N_A \sqrt{\frac{1}{2\pi RT M_{CH_4}}} p(CH_4) \exp\left(-\frac{E_{ads}}{RT}\right) \quad (S9)$$

where E_{ads} is the adsorption barrier and the pre-exponential factor corresponds to the number of methane molecules hitting the surface of unit area per second. This factor follows from the kinetic theory of ideal gases.

The desorption of methane can be considered as a first-order reaction and its rate can be therefore calculated as

$$r_{des} = \nu_0 \exp\left(-\frac{E_{des}}{RT}\right) c(CH_4^*) \quad (S10)$$

where E_{des} is the desorption barrier. From the equilibrium condition, one obtains the surface concentration of methane:

$$c(\mathrm{CH}_4^*) = \frac{N_A}{\nu_0}\sqrt{\frac{1}{2\pi RTM_{\mathrm{CH}_4}}}p(\mathrm{CH}_4)\exp\left(-\frac{E_{ads}-E_{des}}{RT}\right) \quad \text{(S11)}$$

Finally, the rate of carbon adatoms production may be expressed as follows:

$$J = \frac{dc(\mathrm{C}^*)}{dt} \approx N_A\sqrt{\frac{1}{2\pi RTM_{\mathrm{CH}_4}}}\exp\left(-\frac{E_{ads}+E_{dis}-E_{des}}{RT}\right)p(\mathrm{CH}_4) \quad \text{(S12)}$$

A combination of the barriers entering the exponent equals the barrier of dissociative adsorption:

$$E_1 = E_{ads} + E_{dis} - E_{des} = E_{TS}(\mathrm{CH}_3^* - \mathrm{H}^*) - E\left(\mathrm{CH}_{4(\mathrm{g})}\right) \quad \text{(S13)}$$

From Eq. (S12), one can easily obtain the expression used in the main text:

$$J = k_1 p(\mathrm{CH}_4) \quad \text{(S14)}$$

where k_1 satisfies Eq. (A1) of the main text.

3.2. Attachment rates

To derive rate constants $k_{at}(i)$, we consider the 2D ideal gas of i-th particles. Applying the kinetic theory of ideal gases for the case of two dimensions, one obtains that the number of particles hitting the flake's perimeter P per second can be expressed as follows:

$$j(i) = \frac{P}{4}\sqrt{\frac{2RT}{\pi M_i}}c(i) \quad \text{(S15)}$$

Since we consider ideal hexagonal flakes of graphene, the perimeter may be expressed through the number of C atoms in the flake as

$$P = 4\sqrt{\frac{\sqrt{3}n}{2\rho_s}} \quad \text{(S16)}$$

Taking into account that the energy of the hitting particle should exceed the corresponding attachment barrier for a successful attachment, one finds

$$r_{at}(i) = \sqrt{\frac{\sqrt{3}RT}{\pi\rho_s M_i}}\exp\left(-\frac{E_{at}(i)}{RT}\right)c(i)\sqrt{n} \quad \text{(S17)}$$

which explains Eqs. (3,A3) for $r_{at}(i)$ and $k_{at}(i)$ presented in the main text.

The dimerization rate constant k_d is derived in an analogous way as $k_{at}(i)$ because the number of binary C-C collisions may be calculated by considering the flow of C atoms through the circle of radius $r_d^{\neq}$ centered on another C atom. Additionally, we note that, in contrast with the gas phase kinetics, where binary elastic collisions of atoms of the same kind cannot lead to dimerization, in this case it is possible, since the excess of kinetic energy can be effectively "absorbed" by the vibrational modes of the metal surface (in the gas phase, it requires the participation of the third particle in the collision).

3.3. Detachment rates

Let us consider a hexagonal flake of graphene consisting of n atoms. The total number of C atoms or C_2 fragments on the edges of this flake can be calculated as:

$$N_{edge}(i) = \frac{P}{\delta(i)} = \frac{4}{\delta(i)}\sqrt{\frac{\sqrt{3}n}{2\rho_s}} \quad \text{(S18)}$$

where δ_C and δ_{C_2} are the lengths of the zigzag edge occupied by a single atom or by a C_2 fragment. Their values following from simple geometric considerations are presented in the main text.

The detachment rate of the *i*-th fragment (C or C_2) from the edge may be considered as a first-order reaction, which yields

$$r_{det}(i) = \nu_0 \exp\left(-\frac{E_{det}(i)}{RT}\right) N_{edge}(i) = \frac{4\nu_0}{\delta(i)}\sqrt{\frac{\sqrt{3}}{2\rho_s}} \exp\left(-\frac{E_{det}(i)}{RT}\right)\sqrt{n} \quad \text{(S19)}$$

This explains Eqs. (4,A4) of the main text.

References

1. Blöchl, P.E. Projector augmented-wave method. *Annalen der Physik* **1994**, *50*, 17953–17979.
2. Jónsson, H.; Mills, G.; Jacobsen, K.W. Nudged elastic band method for finding minimum energy paths of transitions. *Classical and Quantum Dynamics in Condensed Phase Simulations* **1998**, 385–404.
3. Li, P.; Li, Z.; Yang, J. Dominant Kinetic Pathways of Graphene Growth in Chemical Vapor Deposition: The Role of Hydrogen. *The Journal of Physical Chemistry C* **2017**, *121*, 25949–25955
4. Wu, P.; Zhang, Y.; Cui, P.; Li, Z.; Yang, J.; Zhang, Z. Carbon Dimers as the Dominant Feeding Species in Epitaxial Growth and Morphological Phase Transition of Graphene on Different Cu Substrates. *Phys. Rev. Lett.* **2015**, *114*, 216102.
5. Chakraverty, B. Grain size distribution in thin films—1. Conservative systems. *Journal of Physics and Chemistry of Solids* **1967**, *28*, 2401–2412.
